# Proteomic and biochemical analyses reveal the activation of unfolded protein response, ERK-1/2 and ribosomal protein S6 signaling in experimental autoimmune myocarditis rat model

**DOI:** 10.1186/1471-2164-12-520

**Published:** 2011-10-20

**Authors:** Joo Hee Chung, Hee Jung Choi, Soo Young Kim, Kwan Soo Hong, Soo Kee Min, Myung Hee Nam, Chan Wha Kim, Young Ho Koh, Jong Bok Seo

**Affiliations:** 1 Seoul Center, Korea Basic Science Institute, Sungbuk-gu, Seoul 136-713, Republic of Korea; 2 BK21 School of Life Sciences & Biotechnology, Korea University, Sungbuk-gu, Seoul 136-701, Republic of Korea; 3 ILSONG Institute of Life Science, Hallym University, 1605-4 Gwanyangdong, Anyang, Gyeonggi-do 431-060, Republic of Korea; 4 Division of MR Research, Korea Basic Science Institute, Cheongwon 363-883, Republic of Korea; 5 Department of Pathology, Hallym Sacred Heart Hospital, Hallym University Medical School, 1605-4 Gwanyangdong, Anyang, Gyeonggi-do 431-060, Republic of Korea

## Abstract

**Background:**

To investigate the molecular and cellular pathogenesis underlying myocarditis, we used an experimental autoimmune myocarditis (EAM)-induced heart failure rat model that represents T cell mediated postinflammatory heart disorders.

**Results:**

By performing unbiased 2-dimensional electrophoresis of protein extracts from control rat heart tissues and EAM rat heart tissues, followed by nano-HPLC-ESI-QIT-MS, 67 proteins were identified from 71 spots that exhibited significantly altered expression levels. The majority of up-regulated proteins were confidently associated with unfolded protein responses (UPR), while the majority of down-regulated proteins were involved with the generation of precursor metabolites and energy metabolism in mitochondria. Although there was no difference in AKT signaling between EAM rat heart tissues and control rat heart tissues, the amounts and activities of extracellular signal-regulated kinase (ERK)-1/2 and ribosomal protein S6 (rpS6) were significantly increased. By comparing our data with the previously reported myocardial proteome of the Coxsackie viruses of group B (CVB)-mediated myocarditis model, we found that UPR-related proteins were commonly up-regulated in two murine myocarditis models. Even though only two out of 29 down-regulated proteins in EAM rat heart tissues were also dysregulated in CVB-infected rat heart tissues, other proteins known to be involved with the generation of precursor metabolites and energy metabolism in mitochondria were also dysregulated in CVB-mediated myocarditis rat heart tissues, suggesting that impairment of mitochondrial functions may be a common underlying mechanism of the two murine myocarditis models.

**Conclusions:**

UPR, ERK-1/2 and S6RP signaling were activated in both EAM- and CVB-induced myocarditis murine models. Thus, the conserved components of signaling pathways in two murine models of acute myocarditis could be targets for developing new therapeutic drugs or methods aimed at treating enigmatic myocarditis.

## Background

Myocarditis is an inflammatory heart disease with a wide variety of symptoms that range from mild dyspnea to cardiogenic death [1]. Although viral infection is thought to be the most common cause of acute myocarditis in humans, myocarditis is also associated with various infections, drugs and toxins [1,2]. Myocarditis is a relatively rare disease, and its incidence has been declining since the 1980s. However, 16% of the casualties of sudden infant death syndrome [3], 62% of peripartum cardiomyopathies [4] and a significant proportion of dilated cardiomyopathies [5] may be caused by myocarditis, which indicate the seriousness of this disease.

To investigate the molecular and cellular etiology underlying myocarditis, two different experimental murine models, Coxsackie viruses of group B (CVB)-induced myocarditis [6] and experimental autoimmune myocarditis (EAM)-induced heart failure [7,8], have been widely used. Even though viral infections are known to account for up to 20 - 40% of myocarditis cases, the exact causes are still unknown [1,9]. Because the exact trigger remains unknown in the majority of myocarditis patients and because myocardial injury appears to contribute to various autoimmune responses [1,2,10], these two murine models are complementary alternatives for studying the molecular and cellular etiologies underlying myocarditis. Recently, the myocardial proteomes of mice with CVB3-induced myocarditis were reported [11]. Thus, we investigated the myocardial proteomes of rats with EAM-induced heart failure to determine the similarities and differences between the two murine models to improve our understanding of the molecular and cellular basis of myocarditis.

In this study, we found that 38 and 29 proteins were significantly up- or down-regulated, respectively, in the myocardial proteomes of EAM rats, including unfolded protein response (UPR) target genes and proteins involved in the generation of precursor metabolites and energy metabolism in mitochondria. In addition, extracellular signal-regulated kinase (ERK)-1/2 and ribosomal protein S6 (rpS6) were also activated in the EAM-induced rat models.

## Results

### Histopathological characterization of experimental autoimmune myocarditis-induced heart failure in the rat

**Figure 1:**
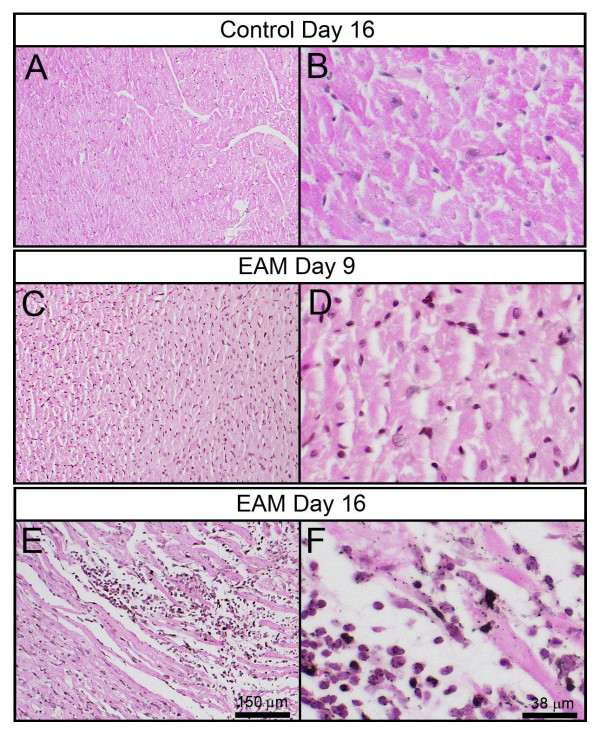
**The histopathological features of myocarditis were recapitulated in the hearts of EAM rats**. Heart sections of control rats (A & B) and EAM day 9 rats (C & D) did not display obvious lymphocyte-rich inflammatory infiltrate. However, myocyte necrosis was observed in the heart sections of EAM day 16 rats (E & F).

One of the pathological criteria for the definition of myocarditis includes the presentation of an interstitial inflammatory cellular infiltrate with necrotic swollen myocytes from hematoxylin/eosin (H&E-stained heart sections [6,7]. Consistent with previous reports, the H&E-stained heart sections from rats 16 days after EAM treatment clearly displayed the presence of numerous mononuclear inflammatory cells within the interstitium and necrotic myocytes (Figures 1E and 1F). These pathological features were not observed in the heart sections of age-matched control rats (Figures 1A and 1B) or rats 9 days after EAM treatment (Figures 1C and 1D). Together with the decreased body weights of the rats 9, 16 and 20 days after EAM (Additional file 1), these pathological results revealed EAM-induced heart failure in the rats.

### Unfolded protein response-related proteins and proteins involved with the generation of precursor metabolites and energy metabolism were among the dysregulated proteins in EAM rat hearts

**Figure 2:**
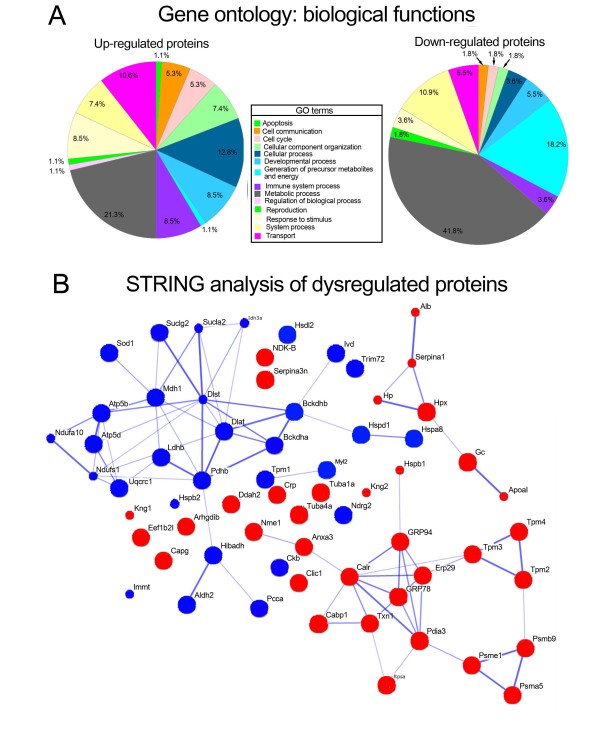
**Gene ontology and interacting gene analysis results**. A. Gene ontology analysis results of up- and down-regulated proteins in EAM rat heart tissue using the PANTHER database [12]. B. Interacting gene analysis results of dysregulated proteins using the STRING database. Nodes with red colors indicate the up-regulated proteins in EAM rat heart tissues compared with those of control rat heart tissues, while the blue colors represent the down-regulated proteins. The size of each node represents the amount of structural information associated with each protein. Confidently associated proteins have pre-calculated links between the two enzymes or proteins in the same metabolic map in the KEGG database, and the width of the lines correlates with the confidence score [13,14].

To identify proteins that were differentially expressed in the hearts of EAM rats, 2-DE-based, unbiased proteomic studies were performed (Additional file 2). For quantitative analyses, the normalized spot volumes of the 2-D gels from EAM rat heart tissues were compared with those of age-matched control rat heart tissues. Thirty-eight spots were significantly up-regulated in the EAM rats compared to the age-matched control rats, while 35 spots were significantly down-regulated (Additional file 3). Only 6 of the 73 spots identified by nano-HPLC-ESI-QIT-MS contained more than 2 proteins (Additional file 3). The lists of proteins with up- and down-regulated spots were loaded into the PANTHER website http://www.pantherdb.org[12] to categorize them according to their biological functions (Table 1, Figures 2A, B, Additional files 4 and 5). Of the 38 up-regulated proteins in the EAM rat heart tissues, 10, 12 and 20 proteins were allocated to transport, cellular processes, and metabolic processes, respectively (Table 1, Figure 2A, Additional file 4). In contrast, of the 29 down-regulated proteins, 6, 10 and 23 proteins were assigned to system processes, the generation of precursor metabolites and energy, and metabolic processes, respectively (Table 1 Figure 2A, Additional file 5). Interestingly, when the dysregulated proteins were combined and analyzed by the Search Tool for the Retrieval of Interacting Genes (STRING; http://www.string-db.org) [13,14], up- or down-regulated proteins, as indicated in Figure 2B by red or blue colored nodes, respectively, showed confidential association only to the same color of nodes, even though several of the red or blue nodes were not linked to any other nodes. Glucose regulated protein (GRP)78, GRP94, endoplasmic reticulum protein 29 (Erp29), protein disulfide isomerase family A, member 3 (Pdia3), calreticulin (Calr), thioredoxin (Txn), calcium-binding protein 1 (Cabp1), proteasome activator complex subunit 1 (Psme1), proteasome subunit beta type-9 (Psmb9), proteasome subunit alpha type-5 (Psma5), tropomyosin beta chain (Tpm2), tropomyosin alpha-3 chain (Tpm3), tropomyosin alpha-4 chain (Tpm4), annexin A3 (Anxa3), heat shock protein beta-1 (Hspb1), nucleoside diphosphate kinase A (Nme1), and 40S ribosomal protein SA (Rpsa) all showed strong associations among the up-regulated proteins (Figure 2B). These strongly associated, up-regulated proteins are known to be involved in unfolded protein responses (UPR). In contrast, the mitochondrial dihydrolipoyllysine-residue succinyltransferase component of the 2-oxoglutarate dehydrogenase complex (Dlst), mitochondrial pyruvate dehydrogenase E1 component subunit beta (Pdhb), branched-chain keto acid dehydrogenase E1 alpha polypeptide (Bckdha), mitochondrial 2-oxoisovalerate dehydrogenase subunit beta (Bckdhb), mitochondrial dihydrolipoyllysine-residue acetyltransferase component of pyruvate dehydrogenase complex (Dlat), succinate-coenzyme A ligase, ADP-forming beta subunit (Sucla2), succinate-CoA ligase, GDP-forming, beta subunit (Suclg2), cytoplasmic malate dehydrogenase (Mdh1), mitochondrial isocitrate dehydrogenase subunit alpha, (Idh3a), mitochondrial ATP synthase subunit delta (ATP5d), mitochondrial ATP synthase subunit beta (ATP5B), heat shock cognate 71 kDa protein (Hspa8), mitochondrial isocitrate dehydrogenase subunit alpha (Idh3a), and Hspb1 all showed strong associations among the down-regulated proteins (Figure 2B). The down-regulated proteins that were classified as mitochondrial proteins have major roles in regulating the generation of precursor metabolites and energy metabolism.

**Table 1:** Allocation of dysregulated proteins in EAM rats according to their biological function

GO Biological process	Name of proteins	**Accession No**.	**Spot No**.	up-regulated (↑) down-regulated (↓)
Apoptosis	Txn	IPI00231368.5	1086	↑
Cell communication	Txn	IPI00231368.5	1086	↑
	Anxa3	IPI00207390.9	638	↑
	Clic1	IPI00421995.1	713	↑
	Arhgdib	IPI00358463.1	837	↑
	Cabp1	IPI00558996.2	136, 148	↑
	Trim72	IPI00361208.4	309	↓

Cell cycle	Txn	IPI00231368.5	1086	↑
	Tuba4a	IPI00362927.1	243	↑
	Tuba1a	IPI00189795.1	243	↑
	Cabp1	IPI00558996.2	136, 148	↑
	Tua1b	IPI00339167.4	243	↑
	Trim72	IPI00361208.4	309	↓

Cellular component organization	Tpm4	IPI00210941.1	684	↑
	Tuba4a	IPI00362927.1	243	↑
	Tpm3	IPI00191354.2	684	↑
	Tpm2	IPI00187731.4	684, 685	↑
	Tuba1a	IPI00189795.1	243	↑
	Capg	IPI00464670.1	477	↑
	Tuba1b	IPI00339167.4	243	↑
	Tpm1	IPI00197888.2	614	↓

Cellular process	Tpm4	IPI00210941.1	684	↑
	Txn	IPI00231368.5	1086	↑
	Anxa3	IPI00207390.9	638	↑
	Tuba4a	IPI00362927.1	243	↑
	Tpm3	IPI00191354.2	684	↑
	Tpm2	IPI00187731.4	684, 685	↑
	Clic1	IPI00421995.1	713	↑
	Tuba1a	IPI00189795.1	243	↑
	Capg	IPI00464670.1	477	↑
	Arhgdib	IPI00358463.1	837	↑
	Cabp1	IPI00558996.2	136, 148	↑
	Tuba1b	IPI00339167.4	243	↑
	Trim72	IPI00361208.4	309	↓
	Tpm1	IPI00197888.2	614	↓

Developmental process	Tpm4	IPI00210941.1	684	↑
	Tuba4a	IPI00362927.1	243	↑
	Tpm3	IPI00191354.2	684	↑
	Tpm2	IPI00187731.4	684, 685	↑
	Tuba1a	IPI00189795.1	243	↑
	Capg	IPI00464670.1	477	↑
	Tuba1b	IPI00339167.4	243	↑
	Ddah2	IPI00215294.1	691	↑
	Trim72	IPI00361208.4	309	↓
	Myl2	IPI00214000.4	968	↓
	Tpm1	IPI00197888.2	614	↓

Generation of precursor metabolites and energy	Txn	IPI00231368.5	1086	↑
	Atp5b	IPI00551812.1	318	↓
	Atp5d	IPI00198620.1	1036	↓
	Ldhb	IPI00231783.5	1112	↓
	Ndufs1	IPI00358033.1	89	↓
	Ivd	IPI00193716.1	464	↓
	Mdh1	IPI00198717.8	580, 599, 1107	↓
	Uqcrc1	IPI00471577.1	356	↓
	Idh3a	IPI00198720.1	509	↓

Immune system process	Txn	IPI00231368.5	1086	↑
	GRP94	IPI00365985.5	53	↑
	GRP78	IPI00206624.1	107	↑
	Crp	IPI00188225.1	763	↑
	Clic1	IPI00421995.1	713	↑
	Hspb1	IPI00201586.1	814	↑
	Kng1	IPI00515829.1	136, 143, 148	↑
	Hp	IPI00382202.2	364, 568, 578, 604, 808	↑
	Hspb2	IPI00213296.1	930	↓
	Hspa8	IPI00208205.1	1108	↓

Metabolic process	Serpina1	IPI00324019.1	859	↑
	Nme1	IPI00194404.5	994	↑
	Atp5b	IPI00551812.1	318	↓
	Hibadh	IPI00202658.1	696	↓
	Atp5d	IPI00198620.1	1036	↓
	Ldhb	IPI00231783.5	1112	↓
	Ivd	IPI00193716.1	464	↓
	Mdh1	IPI00198717.8	580, 599, 1107	↓
	Uqcrc1	IPI00471577.1	356	↓
	Bckdhb	IPI00201636.3	536	↓
	Hspb2	IPI00213296.1	930	↓
	Trim72	IPI00361208.4	309	↓
	Idh3a	IPI00198720.1	509	↓
	Dlst	IPI00551702.2	295, 384	↓
	Ckb	IPI00470288.4	373	↓
	Pcca	IPI00765682.2	105	↓
	Hsdl2	IPI00367240.3	112	↓
	Dlat	IPI00231714.3	167, 173, 183	↓
	Aldh2	IPI00197770.1	295, 297	↓
	Bckdha	IPI00365663.1	383	↓
	Pdhb	IPI00194324.2	1108	↓
	Hspa8	IPI00208205.1	126	↓
	Hspd1	IPI00339148.2	212	↓

Regulation of biological process	Kng1	IPI00515829.1	136, 143, 148	↑

Reproduction	Hp	IPI00382202.2	364, 568, 578, 604, 808	↑
	Trim72	IPI00361208.4	309	↓

Response to stimulus	Txn	IPI00231368.5	1086	↑
	GRP94	IPI00365985.5	53	↑
	GRP78	IPI00206624.1	107	↑
	Crp	IPI00188225.1	763	↑
	Clic1	IPI00421995.1	713	↑
	Hspb1	IPI00201586.1	814	↑
	Kng1	IPI00515829.1	136, 143, 148	↑
	Hp	IPI00382202.2	364, 568, 578, 604, 808	↑
	Hspb2	IPI00213296.1	930	↓
	Hspa8	IPI00208205.1	126	↓

System process	Tpm4	IPI00210941.1	684	↑
	Tpm3	IPI00191354.2	684	↑
	Tpm2	IPI00187731.4	684, 685	↑
	Apoa1	IPI00197703.2	842	↑
	Hspb1	IPI00201586.1	814	↑
	Kng1	IPI00515829.1	136, 143, 148	↑
	Hp	IPI00382202.2	364, 568, 578, 604, 808	↑
	Hspb2	IPI00213296.1	930	↓
	Trim72	IPI00361208.4	309	↓
	Myl2	IPI00214000.4	968	↓
	Ckb	IPI00470288.4	373	↓
	Tpm1	IPI00197888.2	614	↓
	Hsdl2	IPI00367240.3	112	↓

Transport	Hpx	IPI00195516.6	95, 100, 124	↑
	Alb	IPI00191737.6	143	↑
	Gc	IPI00194097.5	254	↑
	Anxa3	IPI00207390.9	638	↑
	Tuba4a	IPI00362927.1	243	↑
	Clic1	IPI00421995.1	713	↑
	Erp29	IPI00207184.1	765	↑
	Tuba1a	IPI00189795.1	243	↑
	Apoa1	IPI00197703.2	842	↑
	Tuba1b	IPI00339167.4	243	↑
	Atp5b	IPI00551812.1	318	↓
	Atp5d	IPI00198620.1	1036	↓
	Trim72	IPI00361208.4	309	↓

### Progressively increased GRP78 and GRP94 in EAM rats

**Figure 3:**
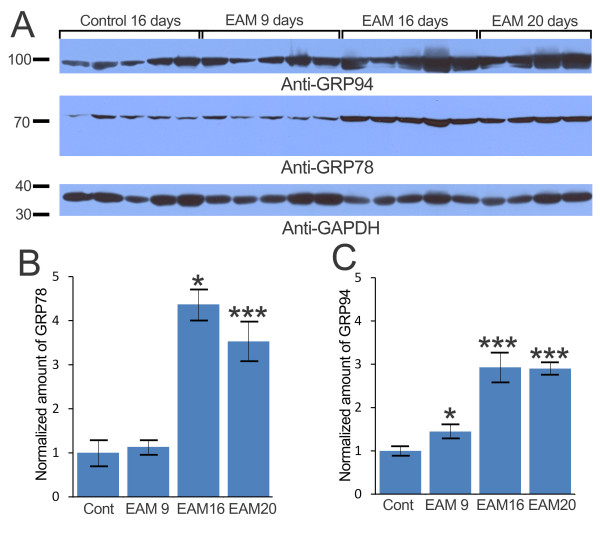
**Progressive increase of GRP78 and GRP94 in EAM hearts**. A. The sample blot was probed with antibodies specific to GRP94, GRP78, and GAPDH. B. The amount of GRP78 was normalized to the loading control, GAPDH. Significantly more GRP78 was present in EAM day 16 and EAM day 20 rats than in the controls. C. The amount of GRP94 was normalized with the loading control, GAPDH. A significantly increased amount of GRP94 was detected in EAM day 9 rats. * indicates *p *< 0.05., *** indicates *p *< 0.001.

The increased expression levels of GRP78 and GRP94 were confirmed via western blot analyses (Figure 3). Compared to control rats, EAM rats showed significantly increased levels of GRP78 and GRP94. On EAM days 16 and 20, rats had 4.37- and 3.53-fold more GRP78, respectively, than the control rats (Figures 3A and 3B). The levels of GRP94 were significantly increased in all EAM rats, i.e., a 1.45-fold increase in EAM day 9 rats, a 2.93-fold increase in EAM day 16 rats and a 2.90-fold increase in EAM day 20 rats (Figure 3C). The confirmation of GRP78 and GRP94 up-regulation together with the proteomic analysis data showing significant increases of spot volumes containing ER stress proteins (Figure 2, Table 1) suggested that UPR may be activated in EAM rats.

### ERK-1/2 and ribosomal protein S6 were activated in EAM rats

**Figure 4:**
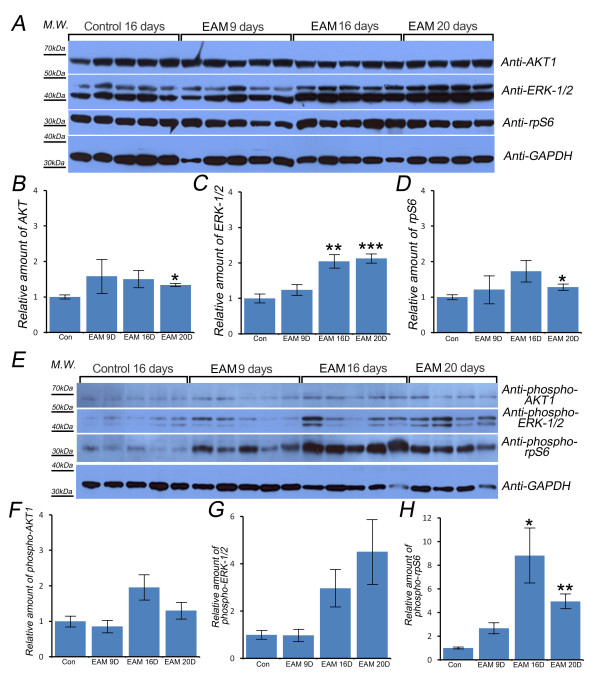
**Total ERK-1/2 and phosphorylated-rpS6 were increased in EAM heart tissue**. The levels of AKT1, ERK-1/2, and rpS6 were examined (A). GAPDH was used as a loading control. There was no difference between the control rats and the EAM day 9 and day 16 rats for AKT1 or rpS6 (B, D). However, the levels of total ERK-1/2 were significantly increased in EAM days 16 and 20 (C). The levels of phosphorylated AKT1, ERK-1/2, and rpS6 were quantified (E). There was no activation of AKT1 signaling in EAM rats (F). EAM day 16 and day 20 rats appeared to have increased amount of phosphorylated ERK-1/2 (G). The levels of phosphorylated rpS6 were significantly increased in EAM day 16 and day 20 rat heart tissues (H). * indicates *p *< 0.05. ** indicates *p *< 0.01. *** indicates *p *< 0.001.

AKT signaling is involved in various cellular processes, including cell survival, growth and metabolism. To examine the role of AKT in EAM pathogenesis, the levels of total and activated AKT were compared (Figure 4). The levels of total and activated AKT1 were not significantly increased, except for total AKT on EAM day 20 (Figures 4B and 4F), which suggested that cell proliferation or apoptosis might not contribute to EAM pathogenesis.

We also examined whether ERK-1/2 signaling, which has been shown to play a role in the susceptibility to CVB3-induced myocarditis in mice, was altered [15]. The levels of total ERK-1/2 were significantly increased in EAM day 16 and day 20 rats (Figure 4C), and the levels of activated ERK-1/2 also appeared to increase in EAM day 16 and day 20 rats (Figure 4F). These results suggested that EAM might increase ERK-1/2 translation and transcription and could contribute to the activation of ERK-1/2.

Because one of the key pathological hallmarks of myocarditis is swollen cytoplasm and nuclei of myocytes with increased translation and transcription [16], and recently, Ruvinsky and his colleagues have shown that the phosphorylation of ribosomal protein S6 (rpS6, Uniprot No. P62755) enhances translation and cell growth *in vivo *[17,18], the levels of total and phosphorylated rpS6 were examined to investigate whether translational efficiency was increased in EAM rats. The total levels of rpS6 were not significantly increased except for EAM day 20 rats (Figure 4D). However, the levels of activated rpS6 were significantly increased in EAM day 16 and day 20 rats (Figure 4H). These results suggested that EAM could increase the translational efficiency of rat heart tissue.

## Discussion

EAM-induced heart failure murine models, which represent a Th17 T cell-mediated postinflammatory heart disease [19], have been widely used to study the molecular and cellular basis of myocarditis pathogenesis [7]. Because the myocardial proteomes of mice with CVB3-induced acute phase myocarditis have recently been reported [11], we investigated the myocardial proteomes of acute phase EAM rats to determine the similarities and differences in dysregulated proteins between the two murine models of myocarditis to improve our understanding of the molecular and cellular basis of myocarditis. Strikingly, of the 39 up-regulated proteins identified in this experiment (Table 1, Figure 2, Additional file 3), UPR target genes, such as GRP78, GRP94, Hspb1, Calr, and Pdia3, were also increased in the acute phase of CVB3-infected myocarditis mouse heart tissues [20]. This result suggests that UPR activation may be a conserved cellular pathogenic mechanism in myocarditis that is caused either by viral infections or autoimmune responses. UPR activation in EAM rat heart tissues was confirmed by western blot analysis, using GRP78- and GRP94-specific antibodies (Figure 3). A more detailed report of the activation of UPR and its consequences in CVB3-induced myocarditis was described in a study demonstrating that the infection of cardiomyocytes with CVB3 activated UPR pathways and induced ER stress-mediated apoptosis [20]. In addition, the treatment of EAM rats with the antioxidant edaravone [21] or the angiotensin II type 1 receptor inhibitor olmesartan [22] has been shown to attenuate ER stress-mediated apoptosis, oxidative stress and cardiac inflammatory mediators. Thus, UPR signaling molecules might be good targets for the development of new therapeutic drugs or treatments aimed at ameliorating or curing enigmatic myocarditis.

Among 29 down-regulated proteins that are mainly associated with the generation of precursor metabolites and energy metabolism in EAM heart tissues (Table 1, Additional file 3), only ATP5b and NADH-ubiquinone oxidoreductase (Ndufs1) were also shown to be dysregulated in CVB-infected myocarditis rat heart tissues. ATP5b and Ndufs1 were significantly increased in the acute phase of CVB-infected myocarditis mouse heart tissues. However, they were significantly decreased during the chronic phase of CVB-infected myocarditis mouse heart tissues [11]. Because other proteins that are known to be involved with the generation of precursor metabolites and energy metabolism in mitochondria, such as ubiquinol-cytochrome c reductase complex core protein, succinate dehydrogenase, succinyl-CoA ligase beta chain, 2-oxoglutarate dehydrogenase E1 component and malate dehydrogenase, were also dysregulated in the CVB-infected myocarditis mouse heart tissues [11], the impaired functions of mitochondria in both murine models of myocarditis may be an underlying common pathogenic mechanism of myocarditis.

In this study, we further tested whether other key cellular and biochemical features of myocarditis were conserved in EAM rat heart tissues. First, we examined whether EAM-induced hypertrophy occurs in cardiomyocytes, as a known cellular feature of myocarditis is an increased heart size with swollen cytoplasm and nuclei in the cardiomyocytes [1,2]. Consistent with this feature, EAM rat heart tissues did have similar levels of total and activated AKT1 compared to the control rat heart tissues (Figures 4A, B, and 4F). Next, we tested whether ERK-1/2 signaling was activated in EAM rats because ERK-1/2 signaling has been shown to be involved in mouse susceptibility to CVB3-induced myocarditis [15]. Interestingly, EAM rat heart tissues had higher levels of activated ERK-1/2, with significantly increased levels of total ERK-1/2 (Figures 4A, C, and 4G). Because the nuclear localization of activated ERK-1/2 is known to be prolonged by oxidative toxicity [23], EAM-induced oxidative stress could contribute to the prolonged localization of activated ERK-1/2 in the swollen nuclei of cardiomyocytes, which would result in increased levels of total and activated ERK-1/2 in the cardiomyocytes. Finally, we tested whether EAM rats had significantly increased levels of activated rpS6 because human patients with post-myocarditis show nuclear hypertrophy with an increased translation efficiency [16], and recent studies have shown that activation of rpS6 enhances translation and cell growth *in vivo *[17,18]. Indeed, EAM rat heart tissues contained increased levels of phosphorylated rpS6 (Figures 4A, E, and 4H), which suggested that the mammalian target of rapamycin and P70-S6 kinase signaling might be activated in EAM rat heart tissues.

## Conclusions

UPR, ERK-1/2, and S6RP signaling we similarly activated in the two murine models of myocarditis. Thus, the development of new therapeutic drugs and treatments should be aimed at deactivating UPR, ERK-1/2, and S6RP signaling.

## Methods

### Experimental autoimmune myocarditis rats

EAM was induced in 7-week-old male Lewis rats as described in Kodama et al. [7] and Smith [8]. Briefly, on days 0 and 6, 1 mg (0.1 ml) of porcine heart myosin, along with an equal volume of complete Fruend's adjuvant, was injected into the rear footpads of the 14 rats. On day 1, the rats were intraperitoneally injected with 500 ng of *Bordetella pertussis *toxin. Heart tissues were obtained on days 9 (5 hearts), 16 (5 hearts) and 20 (4 hearts) after the primary injection. Six control rats were injected without porcine heart myosin or *Bordetella pertussis *toxin. The body and heart weight of rats were measured at each time point. All experimental procedures for this study were performed in accordance with the guidelines of the Animal Ethics of Korea Basic Science Institute (Cheongwon, Republic of Korea).

### Histopathological analysis

Heart ventricular muscle was fixed in 10% formalin and embedded in paraffin. Five-micron thick sections were cut from paraffin-embedded samples and stained using hematoxylin/eosin (H&E). Digital images of sections were taken using an Olympus IX71 camera (Olympus, Tokyo, Japan) and processed using the Adobe Photoshop program (Adobe Systems, San Jose, CA).

### Protein extraction and precipitation

Heart tissue was harvested, immediately washed using cold homogenization buffer A (50 mM Tris-HCl (pH 7.5), 2 mM EDTA, 150 mM NaCl and 0.5 mM DTT) and cut into small pieces. The same heart samples were divided for 2-dimensional polyacrylamide gel electrophoresis analysis or Western blot analysis.

### Two-dimensional polyacrylamide gel electrophoresis

Homogenization buffer B (50 mM Tris-HCl (pH 7.5), 0.25 M sucrose, 5 mM magnesium acetate, 0.2 mM EDTA, 0.5 mM DTT and Halt™ protease inhibitor cocktail (Thermo Fisher Scientific, Rockford, IL)) was added, and tissue samples were homogenized on ice using a sample grinding kit (GE Healthcare Life Science, Uppsala, Sweden). After centrifugation at 13, 000 rpm for 30 min (4°C), 10% trichloroacetic acid was added to the supernatant to precipitate the proteins. The precipitates were redissolved in rehydration buffer (8 M urea, 2% CHAPS, 50 mM DTT and 0.2% IPG buffer) for 2-dimensional polyacrylamide gel electrophoresis. Protein concentrations were determined using a Bradford protein assay kit (Thermo Fisher Scientific).

Two hundred micrograms of protein was separated with an Immobiline Dry Strip (pH 4-7, 18 cm, GE healthcare). The second dimension of the separation was carried out on a 12% acrylamide gel for 7 hr in an Ettan Dalt II system (10 mA/gel; 1 hr, 40 mA/gel; > 6 hr) (GE Healthcare Life Science, Uppsala, Sweden). Gels were stained using a previously described modified silver staining technique [24], and image analysis and spot detection of these gels were performed using Progenesis SameSpots software (version. 4.1, Nonlinear Dynamics, Newcastle, UK). The gel images were aligned by automated calculation of alignment vectors. The automatic analysis was performed on all the aligned images using the analysis wizard. Two of the 6 control gels and one of the 5 EAM rat heart tissue gels exhibited either too few or too many spot numbers and were subsequently excluded. The numbers of spots in the 4 control rat gels were 1469, 981, 1221 and 1394. The numbers of spots in the 4 EAM rat gels were 1633, 1675, 1881 and 1819. The aligned images were grouped to reflect the biological grouping and were used to generate a reference (master) image with 1037 spots using the Progenesis SameSpots software. The reference image was used to normalize and quantify the spot volumes. The list of differentially expressed spots was selected based on *p*-values of ANOVA (*p *< 0.05).

### Nano-HPLC-ESI-QIT-MS and protein identification

For MS analysis, we modified previously described methods [25]. Briefly, spots of interest were cut from the gel and digested with trypsin. Protein identification was performed using a nano LC/MS system consisting of a Surveyor HPLC system (Thermo Scientific, Waltham, MA) and electrospray ionization (ESI)-quadrupole ion trap (QIT) mass spectrometer (LCQ Deca XP-Plus, Thermo Finnigan, San Jose, CA, USA) equipped with a nano-ESI source. Ten microliters of tryptic peptides were loaded by the auto sampler onto a C18 trap column (i.d. 300 μm, length 5 mm, particle size 5 μm; LC Packings, Amsterdam, Netherlands) for desalting and concentration at a flow rate of 20 μl/min. The trapped peptides were then back-flushed and separated on a homemade C18 reversed-phase capillary column (75 μm silica tube, length 150 mm, particle size 5 μm). The pump flow rate was split 1:100 for a column flow rate of 150 μL/min. Mobile phase A was 0.5% acetic acid and 0.02% formic acid in water, and B was 0.5% acetic acid and 0.02% formic acid in 80% acetonitrile. Samples were introduced into the column and eluted with a gradient of 5-5-20-50-60-80-100% of mobile phase B for 0-15-18-50-55-60-62 min, respectively. MS and MS/MS spectra were obtained using a heated capillary temperature of 220°C, an ESI voltage of 2.5 kV, and a collision energy setting of 35%. Data-dependent peak selection of the three most abundant ions in the mass spectra was used. Dynamic exclusion was enabled with a maximum repeat count of two, a repeat duration of 0.5 min, and a 3 min exclusion duration. MS/MS mass peak lists were analyzed for *b *and *y *ions using SEQUEST (version 3.3.1, Thermo Finnigan, San Jose, CA) software. SEQUEST was used for the identification of proteins using the IPI RAT database (version 3.59). The SEQUEST results were filtered using the following parameters: a mass tolerance of 2.0 Da for the precursor ion and 1.0 Da for the fragment ions, one missed cleavage per peptide was allowed, and modifications of proteins were not taken into account. The validity of peptide/spectrum matches was assessed using the SEQUEST defined parameters, the cross-correlation score (X_cor_), and the normalized difference in cross-correlation scores, Matched peptide sequences were required to pass the following filters for identification: 1) the uniqueness scores of the matches' normalized difference in cross-correlation scores were at least 0.1, and 2) minimum X_cor _values ≥ 1.90, ≥ 2.20, ≥ 3.75 for singly, doubly, and triply charged ions, respectively.

### Western blot analysis

Fresh heart tissue samples were ground in RIPA buffer (20 mM HEPES, 1% Triton X-100, 1% deoxycholate, 0.1% SDS, 150 mM NaCl, pH 7.2). After centrifugation at 13, 000 rpm for 30 min (4°C), the supernatant was used to quantify the protein concentration using a BCA assay kit (Thermo Fisher Scientific). Approximately 30 μg of protein extracts in RIPA buffer was separated with 12-15% SDS-PAGE, and it was then transferred to a nitrocellulose membrane. Anti-GRP78 antibody (1:1000, BD Biosciences, Franklin Lakes, NJ, USA), anti-GRP94 antibody (1:5000, Abcam, Cambridge, MA, USA), anti-glyceraldehyde 3-phosphate dehydrogenase (GAPDH) antibody (1:1000, Santa Cruz Biotechnology, Santa Cruz, CA, USA), anti-ERK-1/2, anti-phospho-ERK-1/2, anti-Akt1, anti-phospho-Akt1, anti-ribosomal protein S6 (rpS6), and anti-phospho-rpS6 antibodies (1:1000, Cell Signaling Technology, Danvers, MA, USA) were used to quantify the amounts of their specific antigens. The digital images of the developed X-ray films using western blotting luminal reagent (SC-2048, Santa Cruz Biotech., Santa Cruz, CA, USA) by scanning them (HP Scanjet 4850, Hewlett-Packard Development Company, L.P., Palo Alto, CA, USA) and saving them as a TIFF file. All band intensities in the digital image files were semi-automatically measured using the "wander tools" and "histogram" functions in Adobe Photoshop (Adobe), and the intensities were normalized to the GAPDH band intensity, as previously described [26]. Minitab software (Minitab, State College, PA, USA) was used to perform the two-sample *t*-test.

## Abbreviations

nano-HPLC-ESI-QIT-MS: nano-high performance liquid chromatography-electron spray ionization-quadrupole ion trap-mass spectroscopy.

## Authors' contributions

GSH generated the EAM rat model, and SKM performed the histological analysis. JHC, HJC, SYK, MHM, and CHW acquired data. YHK and JBS analyzed the data and wrote the manuscript. JHC and HJC should be considered as joint first authors. All authors read and approved the final manuscript.

## Supplementary Material

Additional file 1 **Body weight changes in rats with EAM**. The mean body weight of EAM rats was compared to that of age-matched control rats. Controls were sacrificed at day 16, and the mean body weight of EAM day 20 rats was compared to that of day 16 control rats. Click here for file

Additional file 2 **The reference 2-DE gel image of EAM rat heart tissue**. The proteins were separated using IEF (pH 4-7, 18 cm) and 12% SDS-PAGE. Gels were stained with silver nitrate. Blue and red arrows or numbers indicate decreased and increased spots, respectively. Click here for file

Additional file 3 **Significantly increased/decreased spots and identified proteins**. Click here for file

Additional file 4 **Gene ontology analysis results of significantly up-regulated proteins in EAM rat heart tissue**. Click here for file

Additional file 5 **Gene ontology analysis results of significantly down-regulated proteins in EAM rat heart tissue**. Click here for file
