# Supplementary figures and images for: Proteomic and biochemical analyses reveal the activation of unfolded protein response, ERK-1/2 and ribosomal protein S6 signaling in experimental autoimmune myocarditis rat model
Source: BMC Genomics. 2011 Oct 20;12:520. doi: 10.1186/1471-2164-12-520 (PMC3209477; doi:10.1186/1471-2164-12-520)

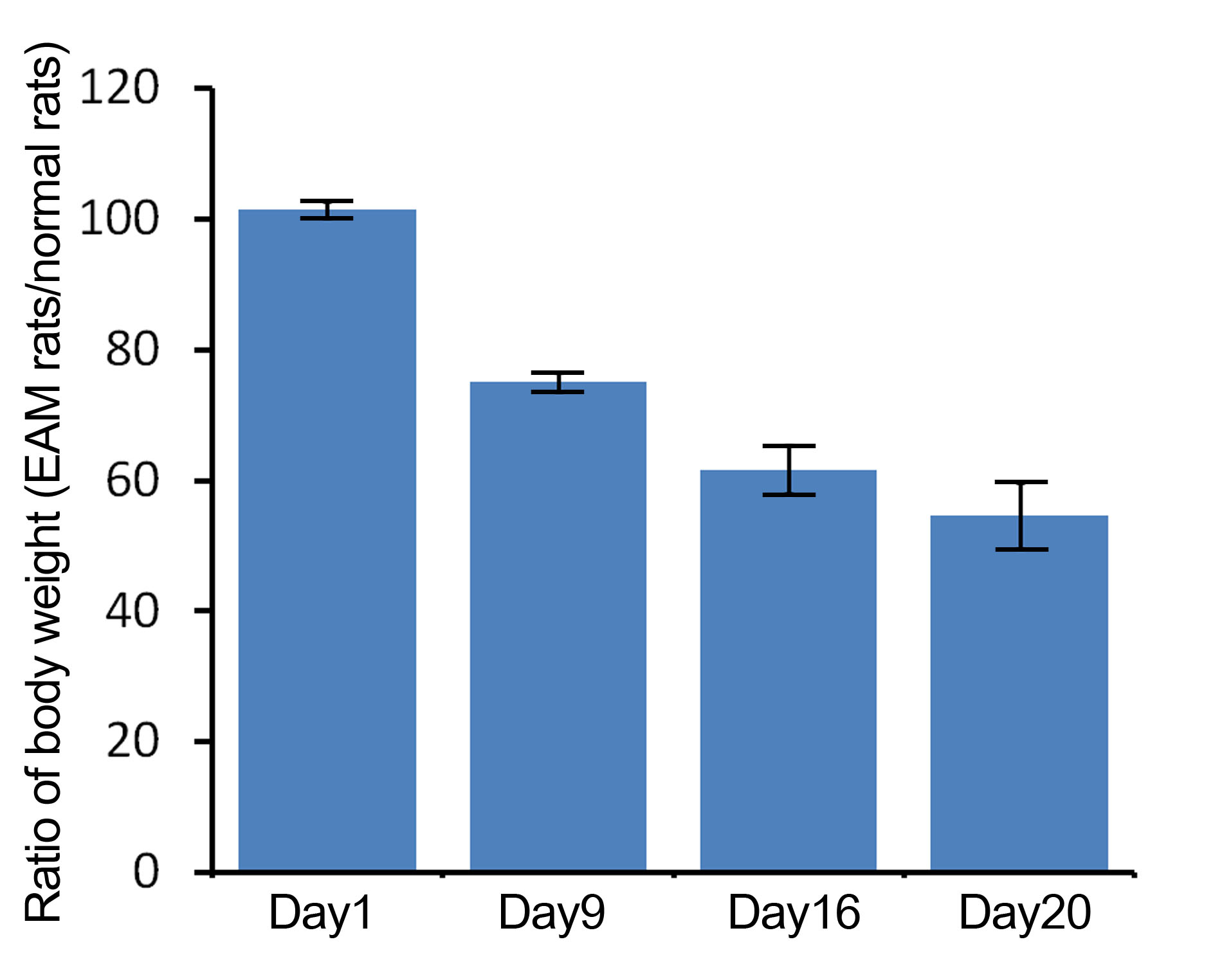

Supplement: Additional file 1 — Body weight changes in rats with EAM. The mean body weight of EAM rats was compared to that of age-matched control rats. Controls were sacrificed at day 16, and the mean body weight of EAM day 20 rats was compared to that of day 16 control rats. [file 1471-2164-12-520-S1.JPEG]

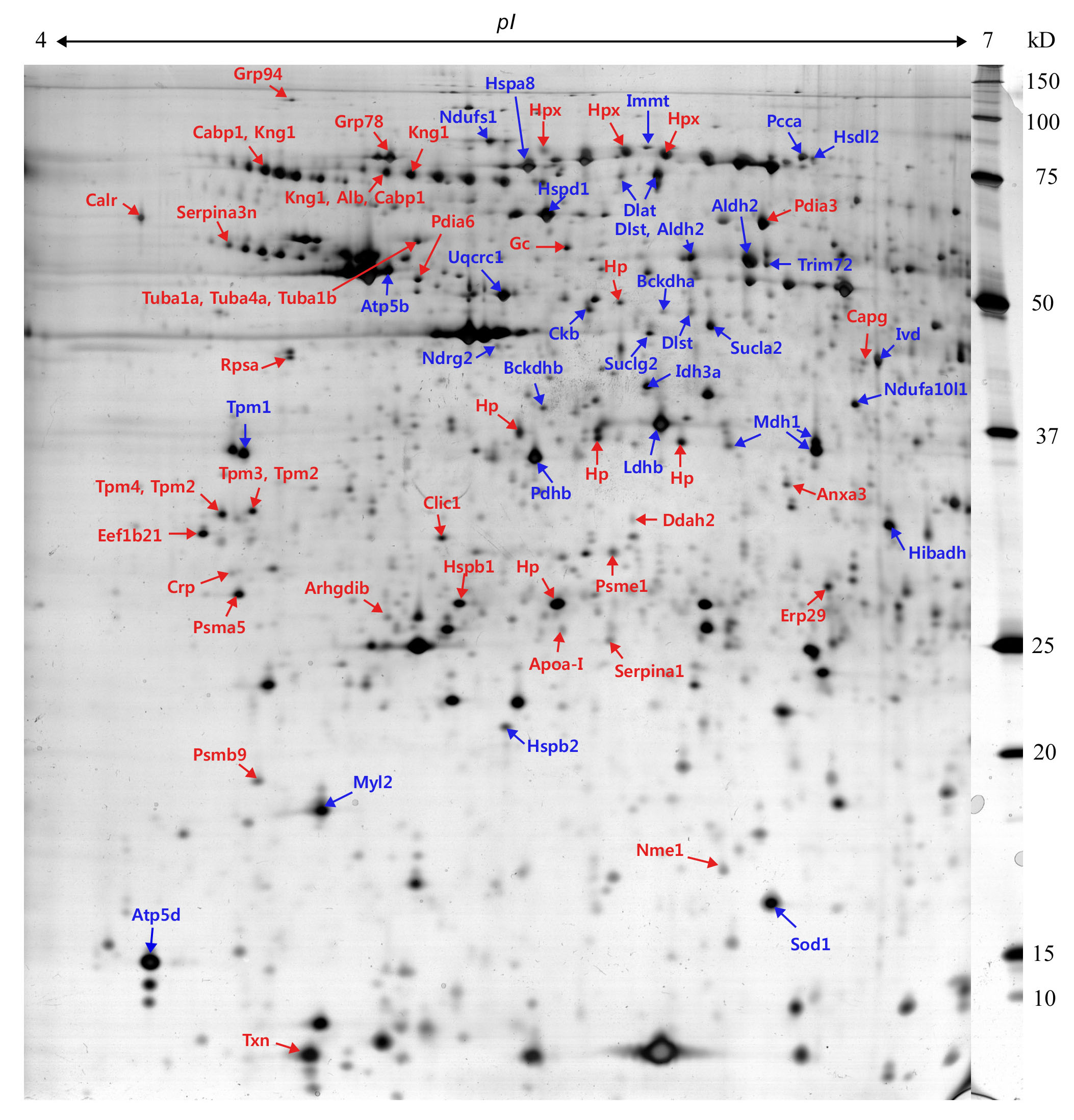

Supplement: Additional file 2 — The reference 2-DE gel image of EAM rat heart tissue. The proteins were separated using IEF (pH 4-7, 18 cm) and 12% SDS-PAGE. Gels were stained with silver nitrate. Blue and red arrows or numbers indicate decreased and increased spots, respectively. [file 1471-2164-12-520-S2.JPEG]
