# Supplementary material for: Proteomic and biochemical analyses reveal the activation of unfolded protein response, ERK-1/2 and ribosomal protein S6 signaling in experimental autoimmune myocarditis rat model
Source: BMC Genomics. 2011 Oct 20;12:520. doi: 10.1186/1471-2164-12-520 (PMC3209477; doi:10.1186/1471-2164-12-520)
Supplement: Additional file 3 — Significantly increased/decreased spots and identified proteins. [file 1471-2164-12-520-S3.DOC]

**Additional file 3**

**Significantly increased/decreased spots and identified proteins**

| Spot  # | Accession No. | Identified protein | Average normalized volumes | | Anova (*p*) | Fold | # of matched peptides / coverage (%) | Molecular weight/PI | ↑↓ |
| --- | --- | --- | --- | --- | --- | --- | --- | --- | --- |
|  |  |  | Control | Myocarditis |  |  |  |  |  |
| 53 | IPI00365985.5 | Isoform 1 of endoplasmin (GRP94, Hsp90b) | 4265.955 | 9951.136 | 0.004873 | 2.33 | 13/13.93 | 92713.25/4.57 | ↑ |
| 89 | IPI00358033.1 | NADH-ubiquinone oxidoreductase 75 kDa subunit, mitochondrial (ndufs1) | 79313.098 | 36480.368 | 0.001355 | 2.17 | 30/32.46 | 79361.7/5.56 | ↓ |
| 95 | IPI00195516.6 | Hemopexin (Hpx) | 10422.466 | 25699.054 | 0.000324 | 2.47 | 9/12.80 | 51318.33/7.43 | ↑ |
| 97 | IPI00364895.4 | 82 kDa protein (Immt) | 37133.707 | 21358.608 | 0.000719 | 1.74 | 6/7.40 | 82197.93/5.29 | ↓ |
| 100 | IPI00195516.6 | Hemopexin (Hpx) | 46817.228 | 88183.915 | 0.001517 | 1.88 | 15/14.60 | 51318.33/7.43 | ↑ |
| 105 | IPI00765682.2 | propionyl-coenzyme A carboxylase, alpha polypeptide (Pcca) | 58967.717 | 30765.703 | 0.009169 | 1.92 | 27/31.30 | 79943.18/6.67 | ↓ |
| 107 | IPI00206624.1 | 78 kDa glucose-regulated protein (GRP78, Hspa5) | 31831.496 | 40613.796 | 0.036932 | 1.28 | 17/25.70 | 72302.52/4.92 | ↑ |
| 112 | IPI00367240.3 | Hydroxysteroid dehydrogenase-like protein 2 (Hsdl2) | 29005.823 | 14460.895 | 0.004160 | 2.01 | 3/6.10 | 58307.05/5.80 | ↓ |
| 124 | IPI00195516.6 | Hemopexin (Hpx) | 48024.895 | 101564.675 | 0.000120 | 2.11 | 10/13.04 | 51318.33/7.43 | ↑ |
| 126 | IPI00208205.1 | Heat shock cognate 71 kDa protein (Hspa8) | 119315.737 | 91914.020 | 0.029940 | 1.30 | 21/18.10 | 70828.2/5.24 | ↓ |
| 136 | IPI00558996.2 | 48 kDa protein (Calcium- binding protein 1, Cabp1) | 10281.005 | 65143.259 | 0.000001 | 6.34 | 11/11.11 | 47897.57/5.97 | ↑ |
|  | IPI00515829.1 | Kininogen 1 (Kng1) |  |  |  |  | 7/10.23 | 47733.68/6.29 |  |
|  | IPI00187799.1 | Isoform HMW of Kng1 |  |  |  |  | 5/2.82 | 70888.87/6.32 |  |
| 143 | IPI00515829.1 | Kininogen 1 (Kng1) | 7544.919 | 49648.419 | 0.000071 | 6.58 | 22/19.30 | 47733.68/6.29 | ↑ |
|  | IPI00558996.2 | 48 kDa protein calcium-binding protein 1 (Cabp1) |  |  |  |  | 1/3.50 | 47897.57/5.97 |  |
|  | IPI00191737.6 | Serum albumin (Alb) |  |  |  |  | 3/5.80 | 68686.2/6.06 |  |
| 148 | IPI00515829.1 | Kininogen 1 (Kng1) | 15434.577 | 77000.426 | 0.000165 | 4.99 | 11/15.35 | 47733.68/6.29 | ↑ |
| 167 | IPI00231714.3 | Dihydrolipoyllysine-residue acetyltransferase component of pyruvate dehydrogenase complex, mitochondrial (Dlat) | 80110.146 | 36338.789 | 0.003053 | 2.20 | 8/10.10 | 67123.58/8.60 | ↓ |
| 173 | IPI00231714.3 | Dihydrolipoyllysine-residue acetyltransferase component of pyruvate dehydrogenase complex, mitochondrial (Dlat) | 173000.706 | 86141.402 | 0.002685 | 2.01 | 16/14.40 | 67123.58/8.60 | ↓ |
| 183 | IPI00231714.3 | Dihydrolipoyllysine-residue acetyltransferase component of pyruvate dehydrogenase complex, mitochondrial (Dlat) | 82108.796 | 40488.415 | 0.000199 | 2.03 | 15/14.60 | 67123.58/8.60 | ↓ |
| 212 | IPI00339148.2 | 60 kDa heat shock protein, mitochondrial (Hsp60, Hspd1) | 179372.960 | 117664.046 | 0.008005 | 1.52 | 40/50.09 | 60917.48/5.83 | ↓ |
| 230 | IPI00324741.2 | Protein disulfide-isomerase A3 (Pdia3) | 38335.812 | 63771.688 | 0.010044 | 1.66 | 34/43.70 | 57043.07/5.83 | ↑ |
| 238 | IPI00211075.1 | Serine protease inhibitor A3N (Serpina3n) | 5835.222 | 21358.839 | 0.000176 | 3.66 | 23/36.60 | 46622.41/5.19 | ↑ |
| 243 | IPI00189795.1 | Tubulin alpha-1A chain (Tuba1a) | 23586.943 | 32670.600 | 0.045278 | 1.39 | 9/21.10 | 50103.65/4.81 | ↑ |
|  | IPI00362927.1 | Tubulin alpha-4A chain  (Tuba4a) |  |  |  |  | 4/5.60 | 49892.41/4.79 |  |
|  | IPI00339167.4 | Tubulin alpha-1B chain  (Tuba1b) |  |  |  |  | 2/3.10 | 50119.64/4.81 |  |
| 254 | IPI00194097.5 | Vitamin D-binding protein  (Gc) | 12028.460 | 23843.458 | 0.000991 | 1.98 | 8/14.50 | 53509.07/5.56 | ↑ |
| 295 | IPI00551702.2 | Dihydrolipoyllysine-residue succinyltransferase component of 2-oxoglutarate dehydrogenase complex, mitochondrial (Dlst) | 133740.456 | 68795.882 | 0.000594 | 1.94 | 7/14.80 | 48894.5/8.87 | ↓ |
|  | IPI00197770.1 | Aldehyde dehydrogenase, mitochondrial (Aldh2) |  |  |  |  | 5/11.00 | 56452.7/6.67 |  |
| 297 | IPI00197770.1 | Aldehyde dehydrogenase, mitochondrial (Aldh2) | 177917.177 | 101110.907 | 0.003288 | 1.76 | 19/27.36 | 56452.72/6.67 | ↓ |
| 309 | IPI00361208.4 | Tripartite motif-containing protein 72 (Trim72) | 27682.776 | 17001.350 | 0.045959 | 1.63 | 4/8.00 | 52797.93/5.84 | ↓ |
| 318 | IPI00551812.1 | ATP synthase subunit beta, mitochondrial (ATP5B) | 170242.451 | 71120.399 | 0.004694 | 2.39 | 13/24.90 | 56318.59/5.06 | ↓ |
| 328 | IPI00365929.1 | Protein disulfide isomerase-associated 6 (Pdia6) | 5638.786 | 17498.878 | 0.000198 | 3.10 | 15/28.10 | 48729.69/4.90 | ↑ |
| 356 | IPI00471577.1 | Cytochrome b-c1 complex subunit 1, mitochondrial precursor (Uqcrc1) | 146114.627 | 73845.200 | 0.004132 | 1.98 | 6/9.60 | 52815.46/5.51 | ↓ |
| 364 | IPI00382202.2 | Isoform 2 of haptoglobin (Hp) | 7515.944 | 20222.523 | 0.001149 | 2.69 | 8/13.40 | 42447.49/6.10 | ↑ |
| 373 | IPI00470288.4 | Creatine kinase B-type (Ckb) | 64309.510 | 45598.972 | 0.002786 | 1.41 | 18/32.00 | 42698.33/5.31 | ↓ |
| 383 | IPI00365663.1 | Branched-chain keto acid dehydrogenase E1, alpha polypeptide (Bckdha) | 24686.285 | 10942.436 | 0.000423 | 2.26 | 9/11.20 | 50636.22/7.74 | ↓ |
| 384 | IPI00551702.2 | Dihydrolipoyllysine-residue succinyltransferase component of 2-oxoglutarate dehydrogenase complex, mitochondrial (Dlst) | 77700.684 | 27827.752 | 0.000014 | 2.79 | 3/7.30 | 48894.45/8.87 | ↓ |
| 398 | IPI00364431.2 | Succinate-coenzyme A ligase, ADP-forming, beta subunit (Sucla2) | 108594.445 | 52776.599 | 0.001499 | 2.06 | 9/27.30 | 50274.26/7.63 | ↓ |
| 423 | IPI00471539.4 | Succinate-CoA ligase, GDP-fomring, beta subunit (RCG56440, Suclg2) | 87491.785 | 48072.655 | 0.000612 | 1.82 | 12/17.51 | 46959.8/7.70 | ↓ |
| 457 | IPI00326606.4 | Isoform 2 of protein Ndrg2 | 27255.869 | 10888.752 | 0.015144 | 2.50 | 3/10.40 | 39245.31/5.20 | ↓ |
| 464 | IPI00193716.1 | Isovaleryl-CoA dehydrogenase, mitochondrial (Ivd) | 173269.827 | 72795.224 | 0.000369 | 2.38 | 14/25.00 | 46405.8/7.82 | ↓ |
| 474 | IPI00215107.3 | 40S ribosomal protein SA (Rpsa) | 11694.634 | 19563.750 | 0.018219 | 1.67 | 8/15.90 | 32803.43/4.65 | ↑ |
| 477 | IPI00464670.1 | Macrophage-capping protein (Capg) | 9371.523 | 18661.044 | 0.021819 | 1.99 | 3/9.50 | 38774.67/6.10 | ↑ |
| 509 | IPI00198720.1 | Isocitrate dehydrogenase [NAD] subunit alpha, mitochondrial (Idh3a) | 102616.973 | 65375.538 | 0.004407 | 1.57 | 6/14.50 | 39588.06/6.49 | ↓ |
| 526 | IPI00189759.1 | NADH dehydrogenase 1 alpha subcomplex 10-like (Ndufa101l1) | 82479.260 | 33524.893 | 0.001864 | 2.46 | 5/15.21 | 40518.61/7.30 | ↓ |
| 536 | IPI00201636.3 | 2-oxoisovalerate dehydrogenase subunit beta, mitochondrial (Bckdhb) | 35650.684 | 16655.772 | 0.003282 | 2.14 | 9/24.36 | 42795.88/6.41 | ↓ |
| 568 | IPI00382202.2 | Isoform 1 of haptoglobin (Hp) | 9526.786 | 24380.346 | 0.000003 | 2.56 | 10/9.20 | 42447.49/6.10 | ↑ |
| 578 | IPI00382202.2 | Isoform 2 of haptoglobin (Hp) | 22860.337 | 55915.578 | 0.002678 | 2.45 | 10/9.40 | 42447.49/6.10 | ↑ |
| 580 | IPI00198717.8 | Malate dehydrogenase, cytoplasmic (Mdh1) | 151342.414 | 67293.370 | 0.002067 | 2.25 | 9/25.70 | 36460.07/6.16 | ↓ |
| 599 | IPI00198717.8 | Malate dehydrogenase, cytoplasmic (Mdh1) | 47183.518 | 24298.397 | 0.002392 | 1.94 | 3/8.70 | 36460.07/6.16 | ↓ |
| 604 | IPI00382202.2 | Isoform 2 of haptoglobin (Hp) | 13049.488 | 36307.652 | 0.000159 | 2.78 | 13/14.4 | 42447.49/6.10 | ↑ |
| 614 | IPI00197888.2 | Isoform 1 of tropomyosin alpha-1 chain (Tpm1) | 84668.291 | 39752.117 | 0.026471 | 2.13 | 2/7.70 | 32660.7/4.54 | ↓ |
| 638 | IPI00207390.9 | Annexin A3 (Anxa3) | 14343.033 | 22837.388 | 0.012204 | 1.59 | 8/24.38 | 36340.59/5.93 | ↑ |
| 684 | IPI00210941.1 | Isoform 2 of tropomyosin alpha-3 chain (Tpm3) | 6193.449 | 23281.891 | 0.000011 | 3.76 | 2/10.50 | 28702.68/4.54 | ↑ |
|  | IPI00187731.4 | Isoform 2 of tropomyosin beta chain (Tpm2) |  |  |  |  | 1/3.50 | 32937.66/4.48 |  |
|  | IPI00191354.2 | 33 kDa protein (Tpm3) |  |  |  |  | 4/15.50 | 33128.81/4.58 |  |
| 685 | IPI00214905.3 | Tropomyosin alpha-4 chain (Tpm4) | 6827.835 | 25743.333 | 0.000031 | 3.77 | 2/9.30 | 28492.48/4.51 | ↑ |
|  | IPI00187731.4 | Isoform 2 of tropomyosin beta chain (Tpm2) |  |  |  |  | 2/6.7 | 32937.66/4.48 |  |
| 691 | IPI00215294.1 | N,N-dimethylarginine dimethylaminohydrolase 2 (Ddah2) | 4075.704 | 10369.146 | 0.016274 | 2.54 | 3/13.7 | 29669.36/5.61 | ↑ |
| 696 | IPI00202658.1 | 3-hydroxyisobutyrate dehydrogenase, mitochondrial (Hibadh) | 115728.347 | 64982.315 | 0.002000 | 1.78 | 6/12.24 | 35279.6/8.52 | ↓ |
| 707 | IPI00372520.1 | Ac2-067 (Eef1b2l) | 24533.292 | 39535.094 | 0.011907 | 1.61 | 2/5.90 | 27952.4/4.12 | ↑ |
| 713 | IPI00421995.1 | Chloride intracellular channel protein 1 (Clic1) | 9505.239 | 26094.855 | 0.000356 | 2.75 | 6/23.7 | 26963.77/4.94 | ↑ |
| 730 | IPI00555312.2 | Proteasome activator complex subunit 1 (Psme1) | 12992.783 | 23375.572 | 0.005314 | 1.80 | 6/24.70 | 29181.25/5.64 | ↑ |
|  | IPI00361908.3 | Similar to protease 28 subunit, alpha (PA28α) |  |  |  |  | 4/11.80 | 31846.7/5.52 |  |
| 763 | IPI00188225.1 | C-reactive protein (Crp) | 4604.301 | 10410.832 | 0.003893 | 2.26 | 3/9.60 | 25451.73/4.75 | ↑ |
| 765 | IPI00207184.1 | Endoplasmic reticulum protein Erp29 | 10239.247 | 22937.810 | 0.005432 | 2.24 | 9/32.70 | 28556.95/6.25 | ↑ |
| 797 | IPI00191502.5 | Proteasome subunit alpha type-5 (Psma5) | 25656.247 | 42171.247 | 0.000070 | 1.64 | 4/17.84 | 26374.17/4.65 | ↑ |
| 808 | IPI00382202.2 | Isoform 2 of haptoglobin (Hp) | 43647.831 | 104823.144 | 0.000149 | 2.40 | 6/16.00 | 42447.5/6.10 | ↑ |
| 814 | IPI00201586.1 | Heat shock protein beta-1 (Hspb1) | 8504.714 | 41881.564 | 0.000465 | 4.92 | 9/38.8 | 22878.61/6.13 | ↑ |
| 837 | IPI00358463.1 | Rho GDP-dissociation inhibitor 1 (Arhgdib) | 3498.071 | 9406.275 | 0.000003 | 2.69 | 6/29.00 | 22869.54/4.82 | ↑ |
| 842 | IPI00197703.2 | Apolipoprotein A-I (APOA-I) | 4636.741 | 11686.347 | 0.002691 | 2.52 | 3/12.00 | 30043.23/5.43 | ↑ |
| 859 | IPI00324019.1 | Alpha-1-antiproteinase (Serpina1) | 9791.024 | 19476.650 | 0.002783 | 1.99 | 7/12.20 | 46106.61/5.65 | ↑ |
| 930 | IPI00213296.1 | Heat shock protein beta-2 (Hspb2) | 36836.515 | 23547.246 | 0.003488 | 1.56 | 9/47.25 | 20334.26/5.16 | ↓ |
| 955 | IPI00196819.5 | Proteasome subunit beta type-9 (Psmb9) | 6529.695 | 21340.437 | 0.000128 | 3.27 | 6/19.6 | 23309.6/4.70 | ↑ |
| 968 | IPI00214000.4 | Myosin regulatory light chain 2, ventricular/cardiac muscle isoform (Myl2) | 156429.619 | 77064.243 | 0.008913 | 2.03 | 9/47.60 | 18868.39/4.68 | ↓ |
| 994 | IPI00191897.4 | Similar to nucleoside diphosphate kinase B (NDK-B) | 5324.966 | 15792.509 | 0.000176 | 2.97 | 2/8.40 | 16179.14/5.94 | ↑ |
|  | IPI00194404.5 | Nucleoside diphosphate kinase A (Nme1) |  |  |  |  | 4/6.60 | 17181.81/5.92 |  |
| 1009 | IPI00231643.5 | Superoxide dismutase [Cu-Zn] (Sod1) | 158287.475 | 101550.674 | 0.016848 | 1.56 | 8/32.47 | 15902.8/5.89 | ↓ |
| 1036 | IPI00198620.1 | ATP synthase subunit delta, mitochondrial (Atp5d) | 266012.705 | 128237.897 | 0.004093 | 2.07 | 1/11.30 | 17584.2/4.98 | ↓ |
| 1086 | IPI00231368.5 | Thioredoxin (Txn) | 125564.221 | 159674.969 | 0.048822 | 1.27 | 5/22.90 | 11666.7/4.64 | ↑ |
| 1103 | IPI00191728.1 | Calreticulin (Calr) | 9784.637 | 27652.738 | 0.003696 | 2.83 | 1/2.60 | 47965.9/4.18 | ↑ |
| 1107 | IPI00198717.8 | Malate dehydrogenase, cytoplasmic (Mdh1) | 160380.904 | 91948.121 | 0.024354 | 1.74 | 16/38.30 | 36460.07/6.16 | ↓ |
| 1108 | IPI00194324.2 | Pyruvate dehydrogenase E1 component subunit beta, mitochondrial (Pdhb) | 191376.642 | 120445.630 | 0.005538 | 1.59 | 30/35.10 | 38957.08/6.21 | ↓ |
| 1112 | IPI00231783.5 | Lactate dehydrogenase B chain (Ldhb) | 355916.465 | 226671.714 | 0.028858 | 1.57 | 48/47.00 | 36589.08/5.65 | ↓ |
